# Supplementary material for: A logical network-based drug-screening platform for Alzheimer’s disease representing pathological features of human brain organoids
Source: Nat Commun. 2021 Jan 12;12:280. doi: 10.1038/s41467-020-20440-5 (PMC7804132; doi:10.1038/s41467-020-20440-5)
Supplement: Supplementary file 1 — Supplementary Information [file 41467_2020_20440_MOESM1_ESM.pdf]

Supplementary Information

(Supplementary Figures, Supplementary Tables, Supplementary Methods,

and Supplementary References)

A logical network-based drug-screening platform for Alzheimer's disease representing pathological features of human brain organoids

Jong-Chan Park [1,2,3,4,‡], So-Yeong Jang [5,‡], Dongjoon Lee [1,3,‡], Jeongha Lee [1], Uiryong Kang [5], Hongjun Chang [5], Haeng Jun Kim [1,3], Sun-Ho Han [1,2,3], Jinsoo Seo [6], Murim Choi [1], Dong Young Lee [7,8,9], Min Soo Byun [10], Dahyun Yi [7], Kwang-Hyun Cho [5,*], and Inhee Mook-Jung [1,2,3,*]

‡Equally contributed first authors

***Corresponding Authors:**

1) Inhee Mook-Jung, Ph.D.

Department of Biochemistry & Biomedical Sciences, Seoul National University College of Medicine, 103 Daehak-ro, Jongno-gu, Seoul 03080, Republic of Korea; Tel: +82-2-740-8245; Fax: +82-2-3672-7352; Email: inhee@snu.ac.kr

2) Kwang-Hyun Cho, Ph.D.

Department of Bio and Brain Engineering, Korea Advanced Institute of Science and Technology (KAIST), 291 Daehak-ro, Yuseong-gu, Daejeon, 34141, Republic of Korea; Tel: +82-42-350-4325; Fax: +82-42-350-4310; E-mail: ckh@kaist.ac.kr

Supplementary Figures

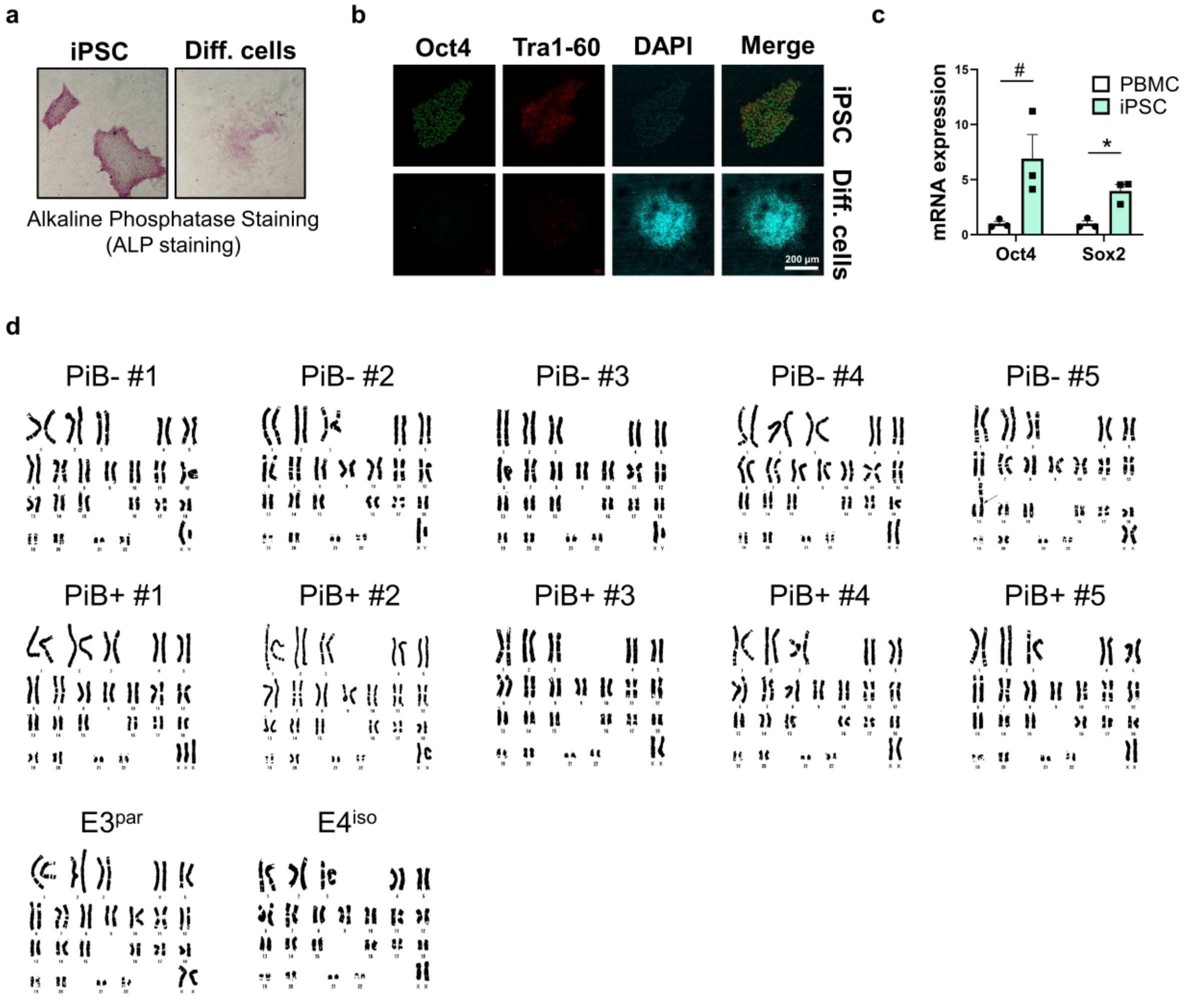

Supplementary Fig. 1. Characterization of generated human iPSCs (a) Alkaline phosphatase staining; iPSCs stained purple color. **(b)** Representative immunocytochemistry images using stem cell- related markers (Oct4, Tra1-60). n = 3 experiments for each iPSC line were repeated independently to check the quality of iPSCs. Scale bar = 200 μm. **(c)** Realtime PCR data showed that iPSCs have higher Oct4, Sox2 mRNA levels than peripheral blood mononuclear cells (n = 3, biologically independent iPSCs; Data are presented as mean values ± standard error of mean, SEM). P-value criteria, $^{\#}p < 0.1$, $*p < 0.05$; $^{\#}p = 0.0547$ for Oct4 and $*p = 0.0105$ for Sox2; unpaired *t*-test (two-sided). Multiple comparison was not applied since Oct4 and Sox2 are independent variables. **(d)** Karyotype analysis. iPSCs, induced pluripotent stem cells; Diff.cells, differentiated cells; PBMC, peripheral mononuclear cells; PiB, Pittsburgh compound B

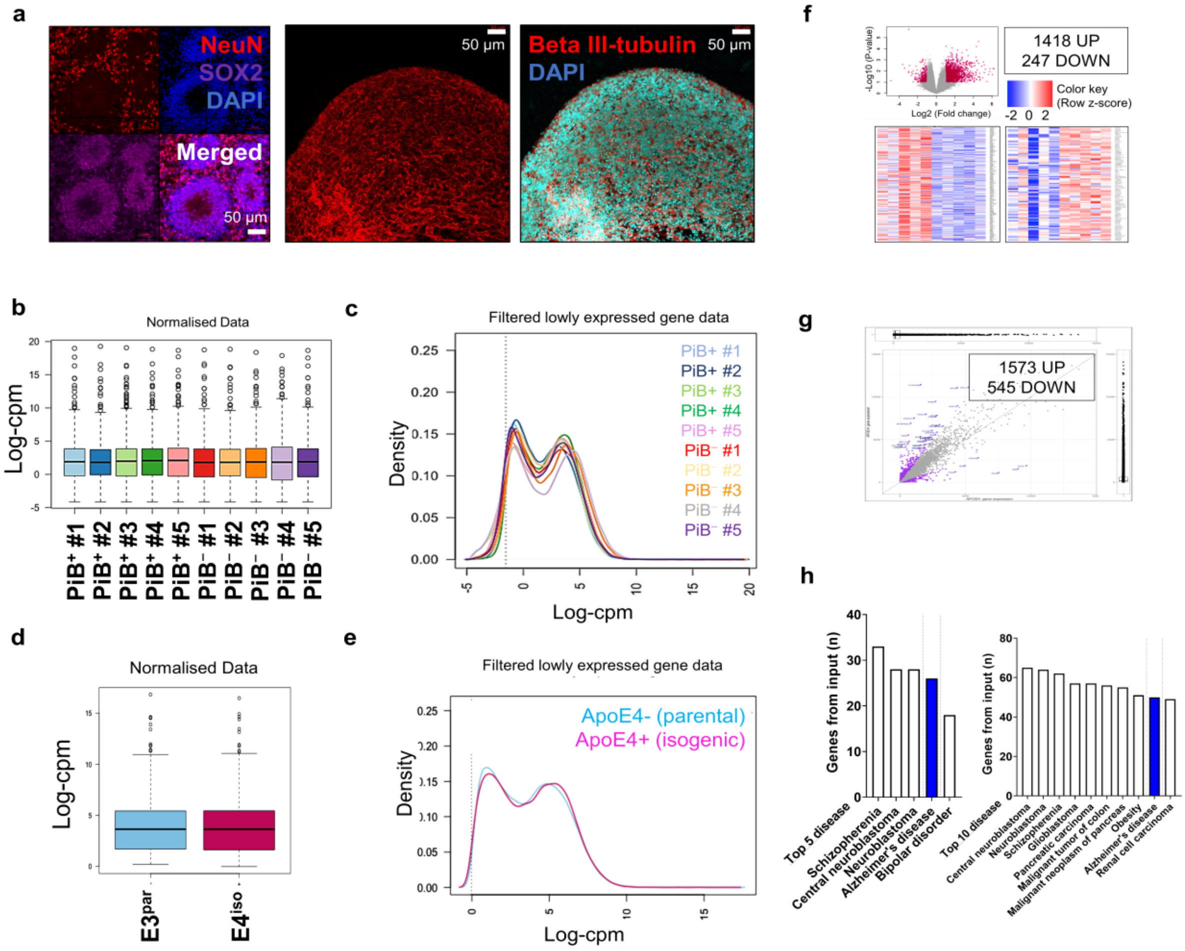

Supplementary Fig. 2. Characterization of generated human iCOs (a) Immunohistochemistry images for the generated iCOs. NeuN, neuronal marker; SOX2, stem cell marker; beta III-tubulin, neuronal marker. n = 3 experiments for each iCO were repeated independently to check the quality of iCOs. White scale bar, 50 μm. **(b)** Gene expression distribution after normalization (box-plots with maximum 3th quartile+1.5IQR of upper whiskers, minimum 1th quartile -1.5IQR of lower whiskers, Q1 25th percentile hinge and Q3 75th percentile hinge for bounds of box, and Q2 50th percentile of median center bold line); n = 10 iCOs were used (n = 1418 genes, up-regulated; n = 247 genes, down-regulated). **(c)** Distribution pattern of gene expression with filtration out lowly expressed genes; Ten iCOs from both PiB^{-} and PiB^{+} were used. **(d)** Gene expression distribution after normalization (box-plots with maximum 3th quartile+1.5IQR of upper whiskers, minimum 1th quartile -1.5IQR of lower whiskers, Q1 25th percentile hinge and Q3 75th percentile hinge for bounds of box, and Q2 50th percentile of median center bold line). **(e)** Distribution pattern of gene expression with filtration out lowly expressed genes (E3^{par} and E4^{iso}) **(f)** Up-regulated (1418 genes) or down-regulated (247 genes) DEG patterns of PiB^{-} and PiB^{+} iCOs. The cut-off was $\log_2$(fold change) > 1 or < -1 and $-\log_{10}$(p-value) > 1. **(g)** Up-regulated (1573 genes) or down-regulated (545 genes) DEG patterns of E3^{par} and E4^{iso} iCOs. The cut-off was $\log_2$(fold change) > 1 or < -1. **(h)** For disease ontology, top 5 diseases (for PiB iCOs) and top 10 diseases (for ApoE iCOs) were selected (FDR- adjusted p-value < 0.1; adjustments were made for multiple comparisons; FDR-

corrected by Toppgene analysis) using the count of ‘genes from input’. Alzheimer’s disease was included in the list. iCOs, iPSC-derived cerebral organoids; Q, quartiles; IQR, Interquartile range;

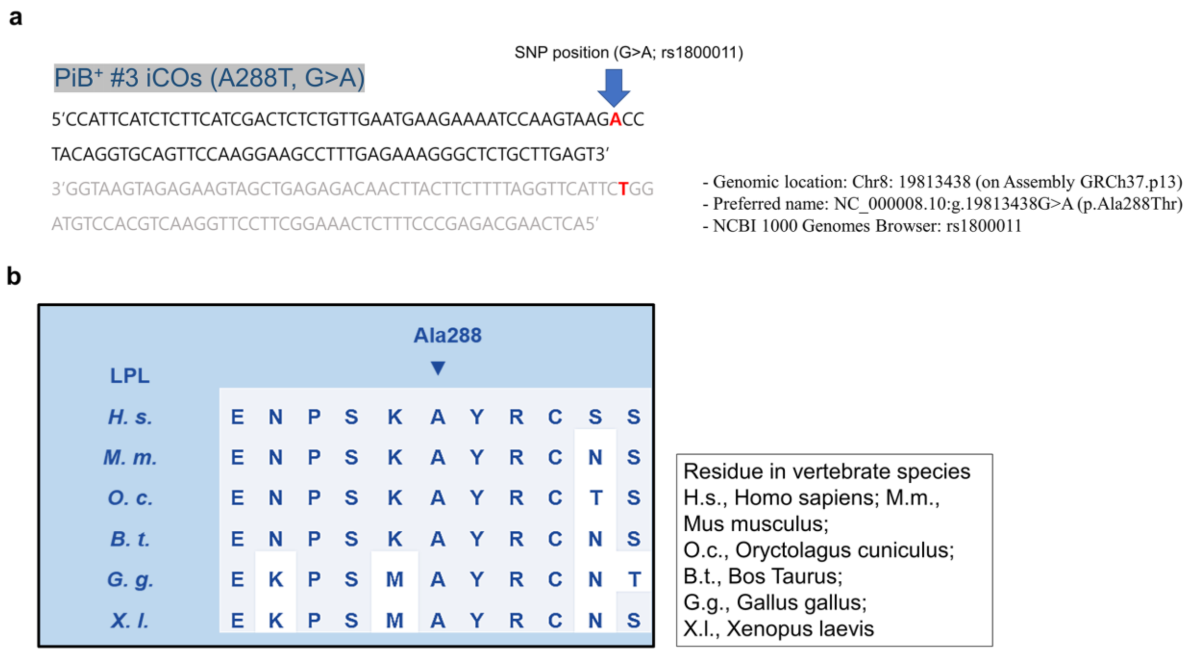

Supplementary Fig. 3. The traits of LPL^{A288T} PiB^{+} #3 iCOs (a) The genomic DNA sequence of PiB^{+} #3 iCO, containing SNP position (G>A; rs1800011) **(b)** The highly conserved sequence of LPL (Ala288) in vertebrate species. SNP, single nucleotide polymorphism; LPL, lipoprotein lipase.

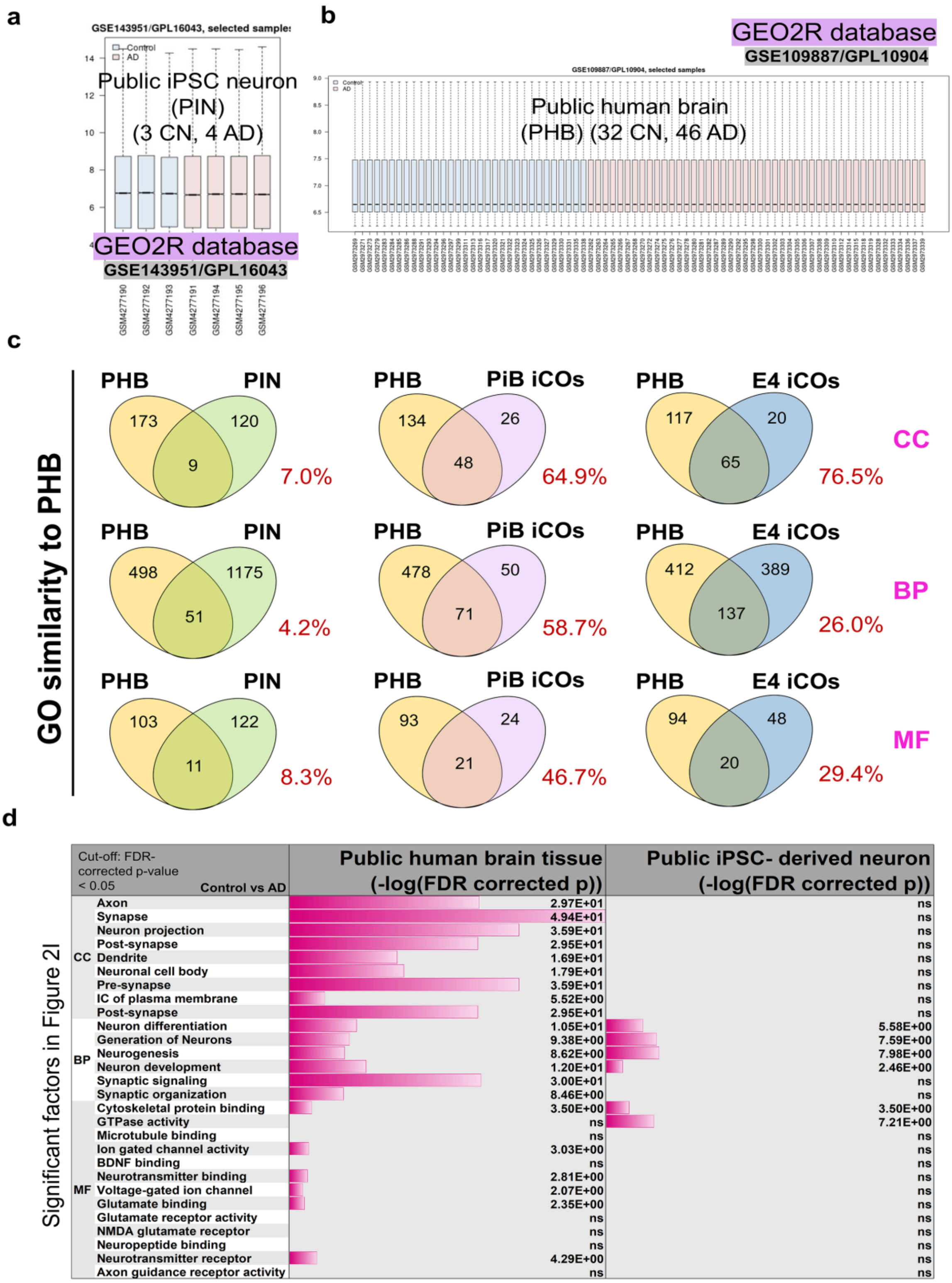

Supplementary Fig. 4. Comparison of transcriptome data from sAD iCOs with GEO2R public database (a-b) Expressed gene distribution (box-plots with maximum 3th quartile+1.5IQR of upper whiskers, minimum 1th quartile -1.5IQR of lower whiskers, Q1 25th

percentile hinge and Q3 75th percentile hinge for bounds of box, and Q2 50th percentile of median center bold line) of iPSC- derived neuron (3 cognitively normal (CN), 4 AD) and human brain (mid-temporal gyrus) transcriptome data (32 CN, 46 AD) from GEO2R public database (Accession number: GSE143951, GSE109887; Platform number: GPL16043, GPL10904). **(c)** Gene Ontology (GO) similarity analysis between public human brain data (PHB) and public iPSC-derived neuron (PIN) or PiB iCOs or $E4^{iso}$ iCOs. Transcriptomic GO analyses were performed with the FDR- adjusted p-value < 0.05, using down-regulated DEGs (cutoff, log_2(fold change) <-1 and $-log_{10}$ (p-value) >1). PHB GO factors were considerably overlapped with those of PiB iCOs and E4 iCOs but not with those of PIN. MF, molecular function; BP, biological process; CC, cellular component. **(d)** -log_{10}(FDR- corrected p-values) of PHB and PIN (cutoff, FDR- adjusted p-value < 0.05). Most of the GO factors in Figure 2I were overlapped with those of PHB but not with those of PIN.

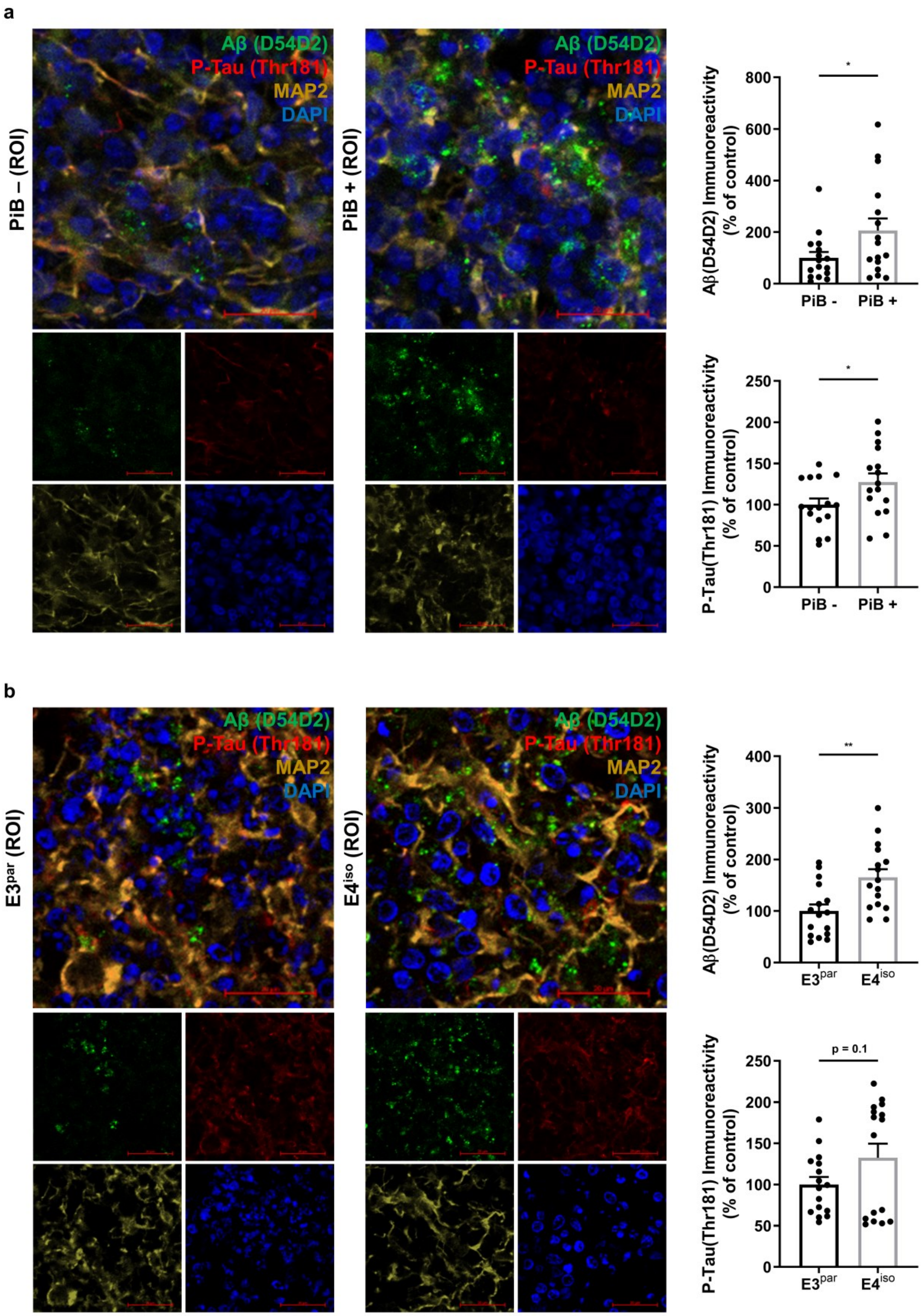

Supplementary Fig. 5. High-resolution (40X) immunohistochemistry images of organoids

(a) Representative 2D organoid images for PiB^{-} iCOs and PiB^{+} iCOs, using Zeiss LSM 700. β-amyloid (green), P-Tau (red), MAP2 (yellow), DAPI (blue), (scale bar, 20 μm). P-value criteria, *$p < 0.05$, **$p < 0.01$; *$p = 0.0492$ and *$p = 0.0414$; two-sided p-values; unpaired *t*-test; n = 16 iCO-slices from were used for each group (from N = 4 PiB^{-} iCOs and N = 4 PiB^{+} iCOs). Data are presented as mean values ± standard error of mean (SEM). **(b)** Representative 2D organoid images for $E4^{iso}$ iCOs and $E3^{par}$ iCOs, using Zeiss LSM 700. β-amyloid (green), P-Tau (red), MAP2 (yellow), DAPI (blue), (scale bar, 20 μm). P-value criteria, *$p < 0.05$, **$p < 0.01$; **$p = 0.0032$ and $p = 0.1066$; two-sided p-values; unpaired *t*-test; n = 16 iCO-slices were used for each group (from N = 4 $E3^{par}$ iCOs and N = 4 $E4^{iso}$ iCOs). Data are presented as mean values ± standard error of mean (SEM). The following antibodies were used: β-amyloid (D54D2) (1:300; 8243, Cell Signaling Technology), phospho-Tau (Thr181) (1:500; MN1050, Thermo Fisher), MAP2 (1:500; ab5392, Abcam), and Alexa Fluor-conjugated secondary antibodies. ROI, region of interests.

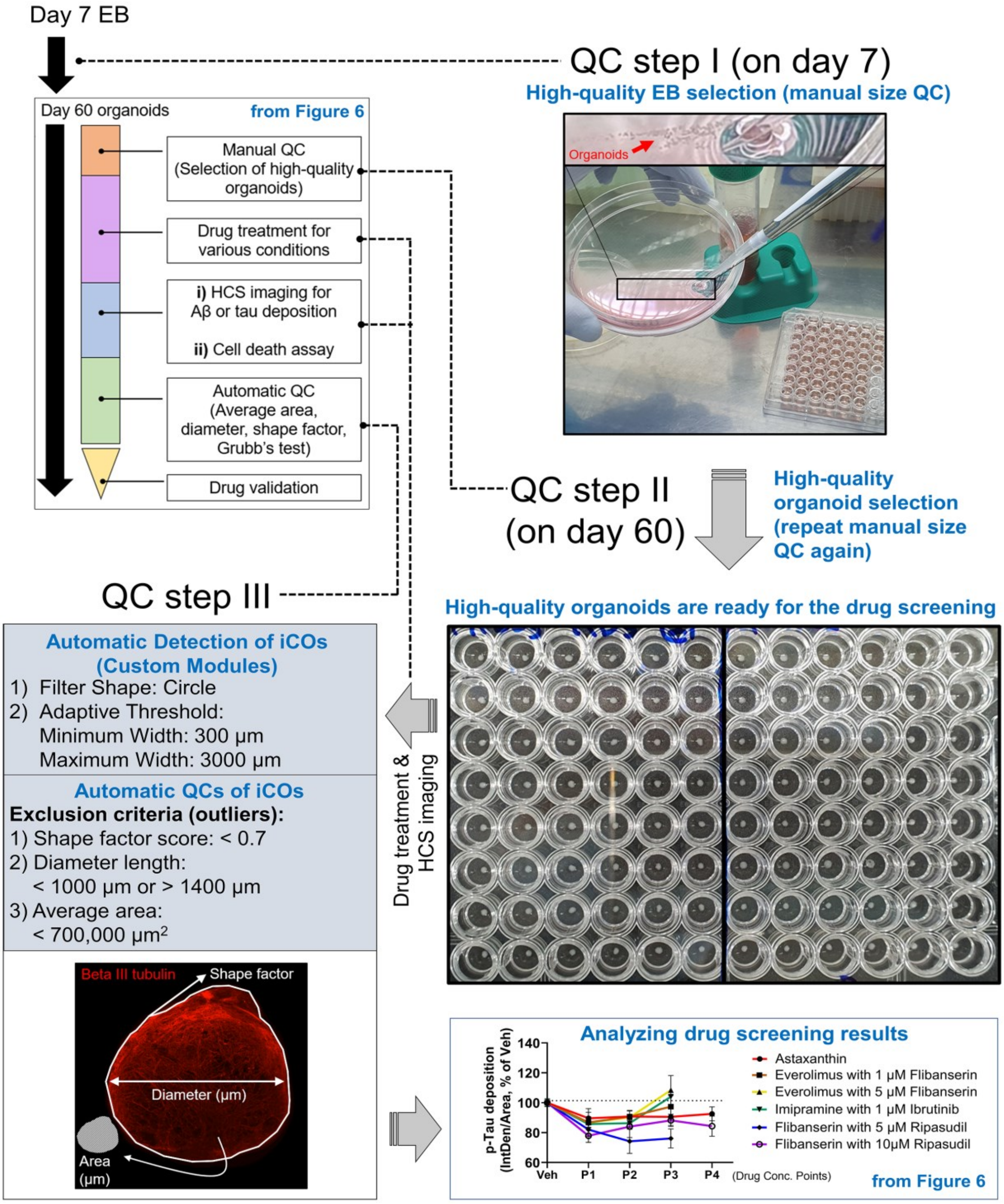

Supplementary Fig. 6. iCO QCs for the HCS system Stepwise QCs were performed to minimize the size-variations and shape-distortion of iCOs. Manual QCs were done twice (day 7 and day 60) and high-quality iCOs were selected. Automatic QCs were done during the drug screening and we applied three exclusion criteria: Shape factor, Diameter length, and Average area. After that, drug screening results were analyzed. QC, quality control; EB, embryoid body; HCS, high-content screening.

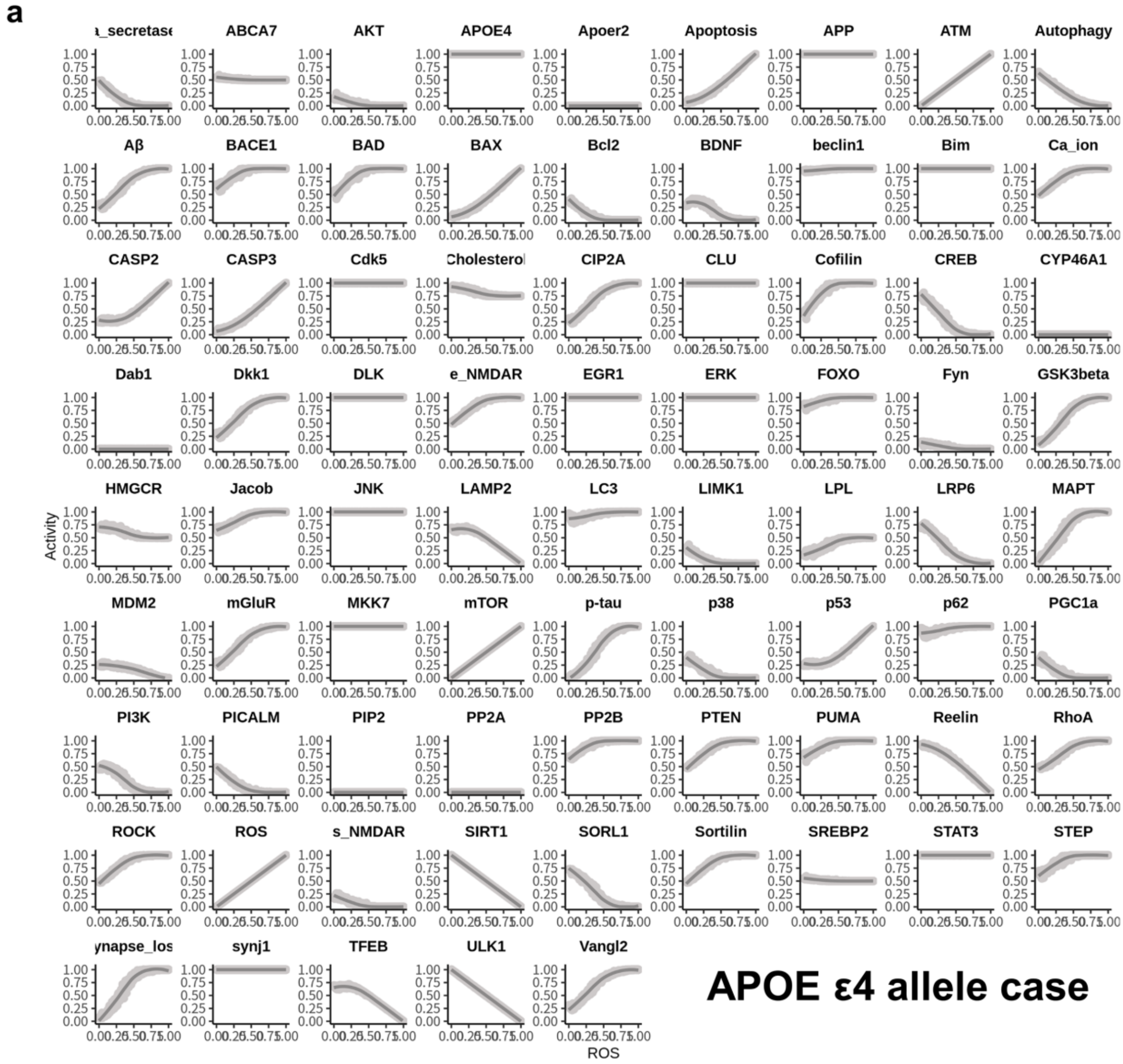

Supplementary Fig. 7-1. Qualitative input-output relationships of the AD molecular regulatory network model for ApoE ε4 allele case Qualitative simulations were performed by varying oxidative stress (ROS) from 0 % to 100 % over one thousand (n > 1000) independent simulations to examine the model's ability (n = 77) to reproduce biological properties of the real AD network with ApoE ε4 allele. Note that the dose-response curves shown here were intended to demonstrate whether the AD network model can qualitatively reproduce the known input–output relationships over a wide range of inputs. Gray solid lines denote 95% confidence intervals around the mean value and each gray blurry area means data-points of independent simulations (error band is so narrow and mostly not distinguishable from the line of mean value). APOE, Apolipoprotein E.

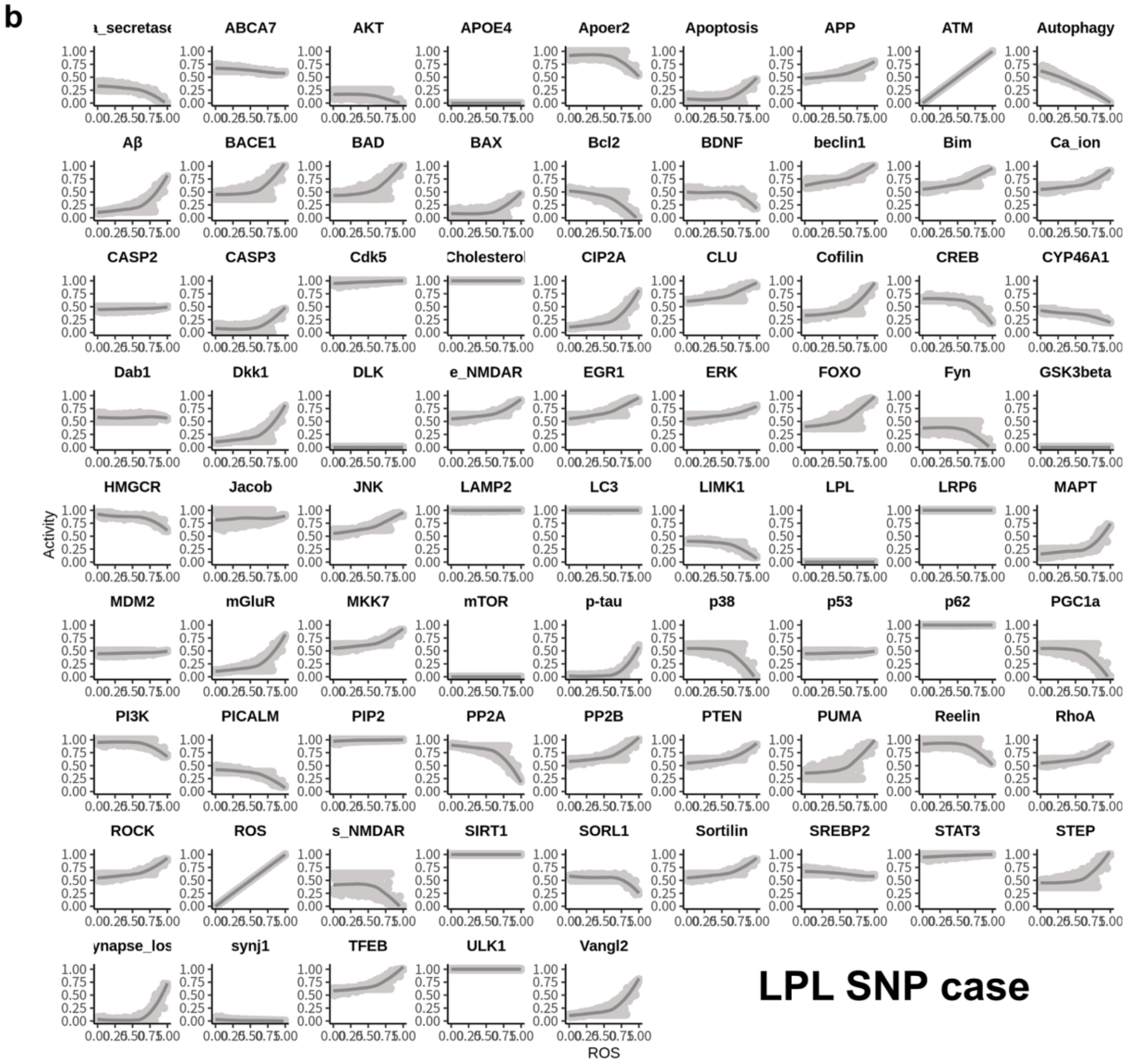

Supplementary Fig. 7-2. Qualitative input-output relationships of the AD signaling network model for LPL SNP case Qualitative simulations were performed by varying oxidative stress (ROS) from 0 % to 100 % over one thousand (n > 1000) independent simulations to examine the model's ability (n = 77) to reproduce biological properties of the real AD network with LPL SNP. Note that the dose-response curves shown here are intended to demonstrate whether the AD network model can qualitatively reproduce the known input–output relationships over a wide range of inputs. Gray solid lines denote 95% confidence intervals around the mean value and each gray blurry area means data-points of independent simulations (error band is so narrow and mostly not distinguishable from the line of mean value). LPL, lipoprotein lipase.

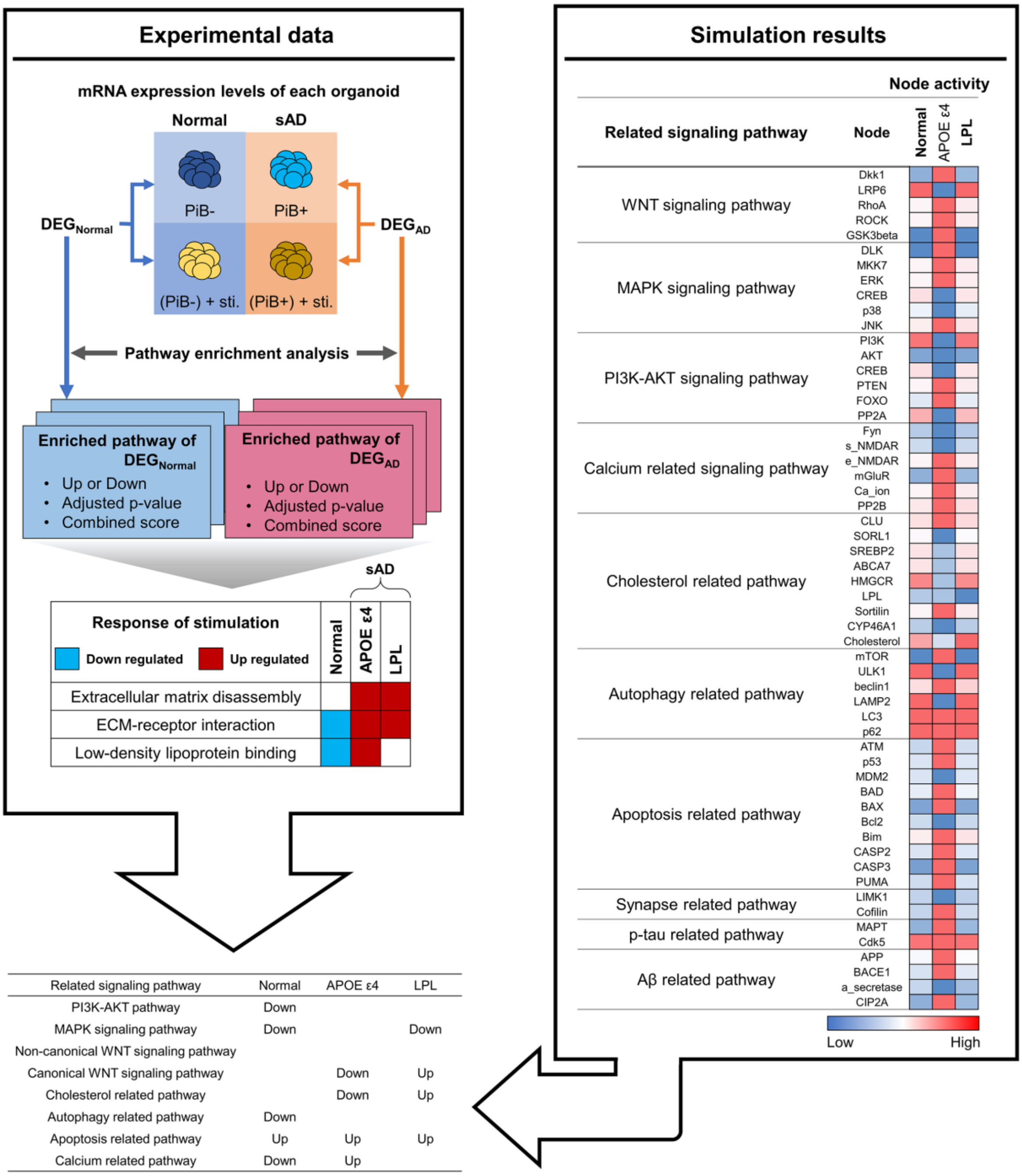

Supplementary Fig. 8. A workflow for model validation using experimental data To validate our model, we have considered aging-related alterations of signaling pathways. Pathway enrichment analysis was performed for differentially expressed genes (DEG) between PiB- iCOs and PiB+ iCOs for normal, ApoE ε4 allele and LPL SNP cases (left). The significantly enriched signaling pathways were compared with the predicted node activities from simulation for each case. DEG analysis and pathway enrichment analysis were performed with DEGseq (FDR-corrected p-value < 0.05, $\log_2$(fold-change) > 1 or $\log_2$(fold-change) <-1) and enrichR (p-value < 0.05), respectively. 'KEGG_2019_Human', 'GO_Molecular_Function_2018', 'GO_Cellular_Component_2018',

‘GO_Biological_Process_2018’, ‘BioCarta_2016’ and ‘Reactome_2016’ DB were used for enriched pathway analysis. These results were compared with the pathway alterations of simulation results.

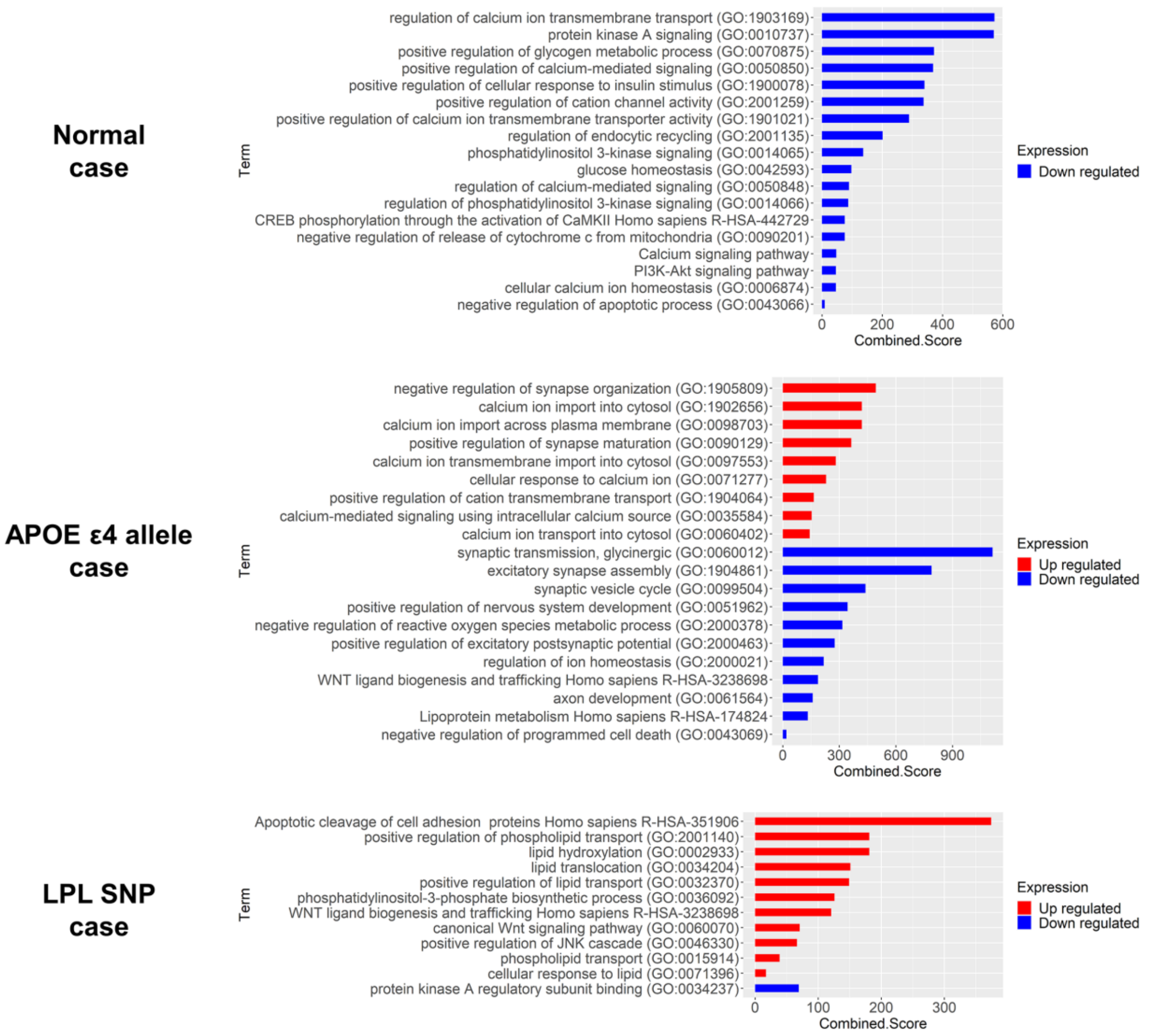

Related signaling pathway	Normal	APOE ε4	LPL
PI3K-AKT pathway	Down		
MAPK signaling pathway	Down		Down
Non-canonical WNT signaling pathway			
Canonical WNT signaling pathway		Down	Up
Cholesterol related pathway		Down	Up
Autophagy related pathway	Down		
Apoptosis related pathway	Up	Up	Up
Calcium related pathway	Down	Up	

Supplementary Fig. 9. Enriched pathway analysis results Enriched pathway analysis of sAD iCOs responses to aging effect (oxidative stress) and the resulting altered signaling pathways (bottom table).

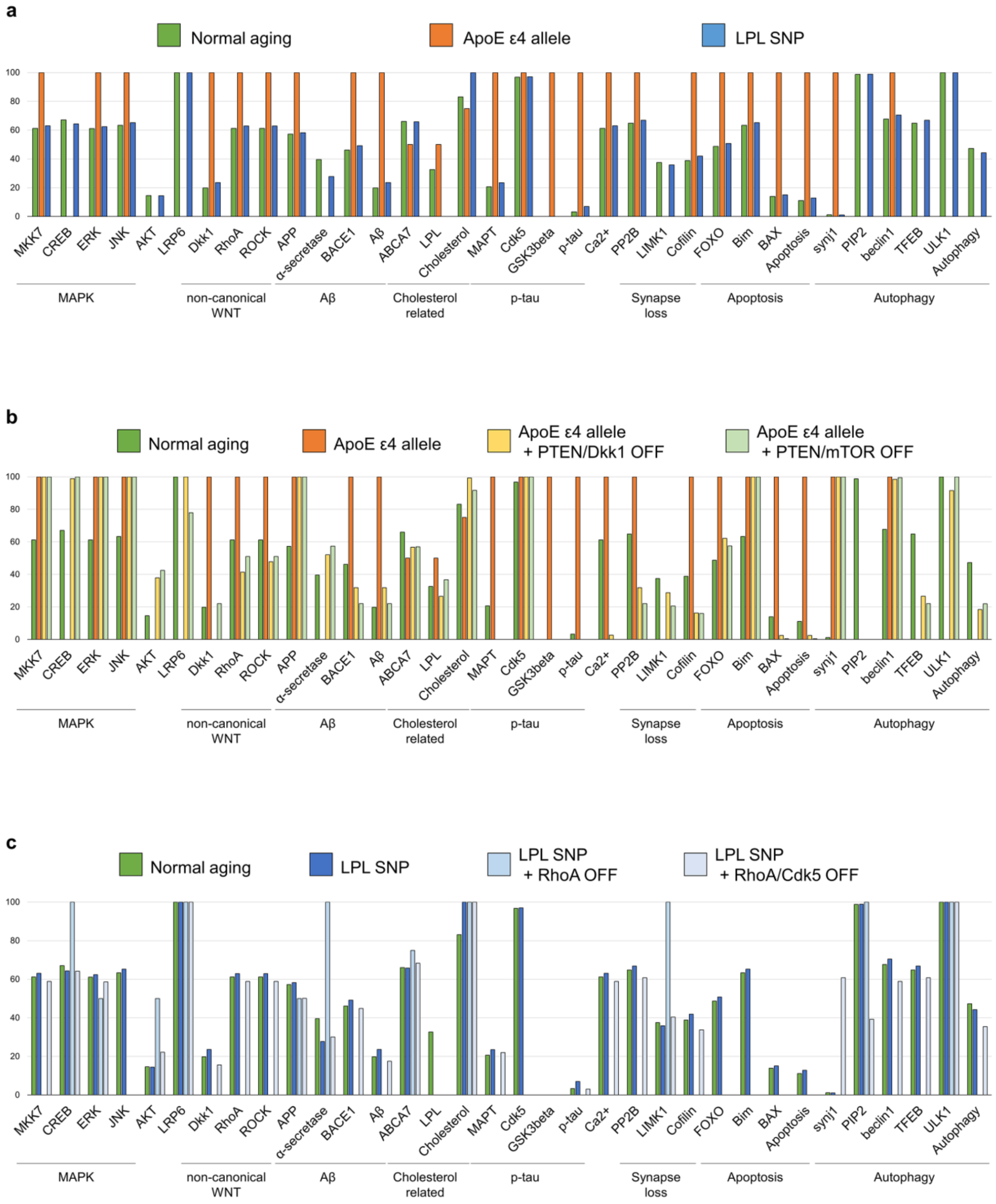

Supplementary Fig. 10. Perturbation analysis results (a) The node activities of major signaling pathways in Normal aging state, ApoE ε4 allele case, and LPL SNP case. **(b)** The perturbation analysis results of ApoE ε4 allele case. **(c)** The perturbation analysis results of LPL SNP case.

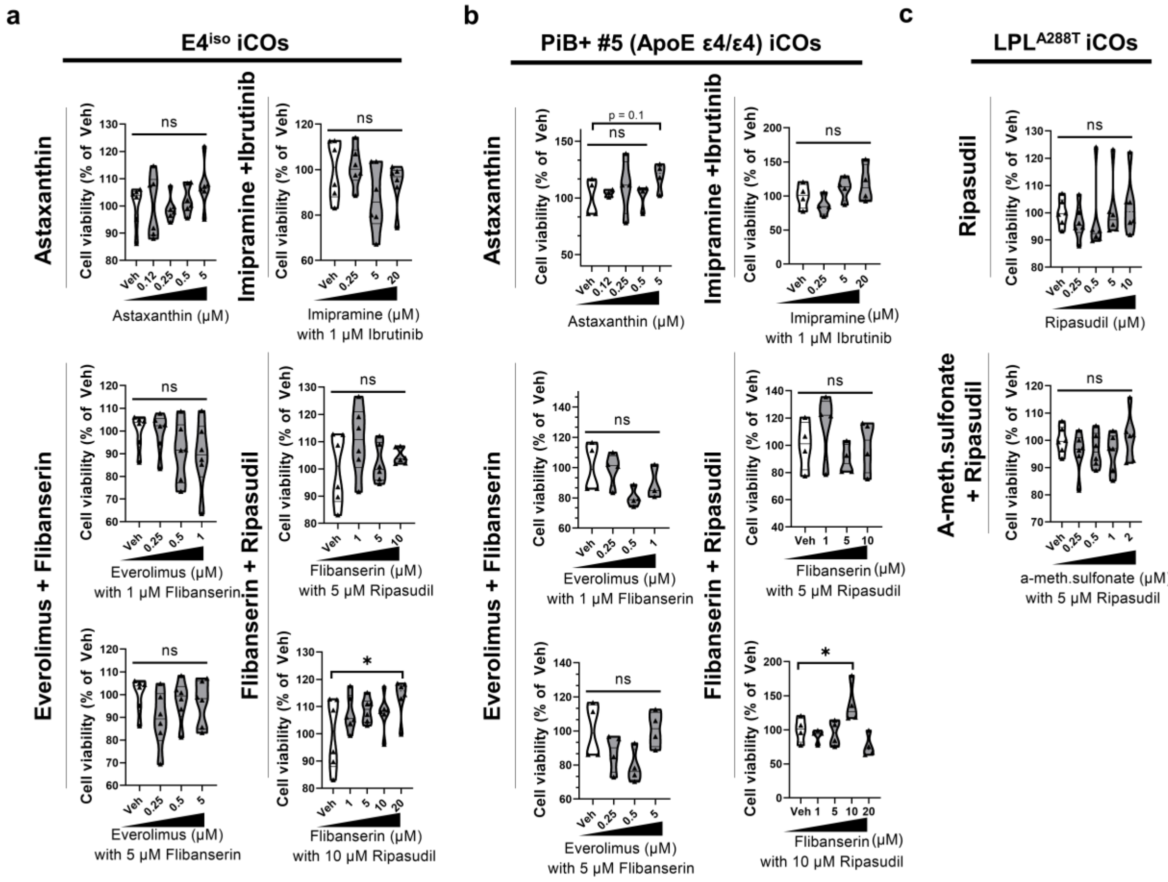

Supplementary Fig. 11. Cell- viability analysis for the sAD iCOs MTT colorimetric assays for the **(a)** $E4^{iso}$ iCOs, **(b)** PiB^{+} iCOs, and **(c)** LPL^{A288T} iCOs. Only Flibanserin with 10 µM Ripasudil showed significant results (*p = 0.0267 for $E4^{iso}$ iCOs, *p = 0.0479 for PiB^{+} #5 iCOs; n = 5 iCOs were used for each drug concentration). P-values from ANOVA with the correction of Tukey post-hoc test for multiple comparisons (cut-off, *p < 0.05). A-meth.sulfonate, Abemaciclib methanesulfonate; MTT, 3-(4,5-dimethylthiazol-2-yl)-2,5-diphenyltetrazolium bromide.

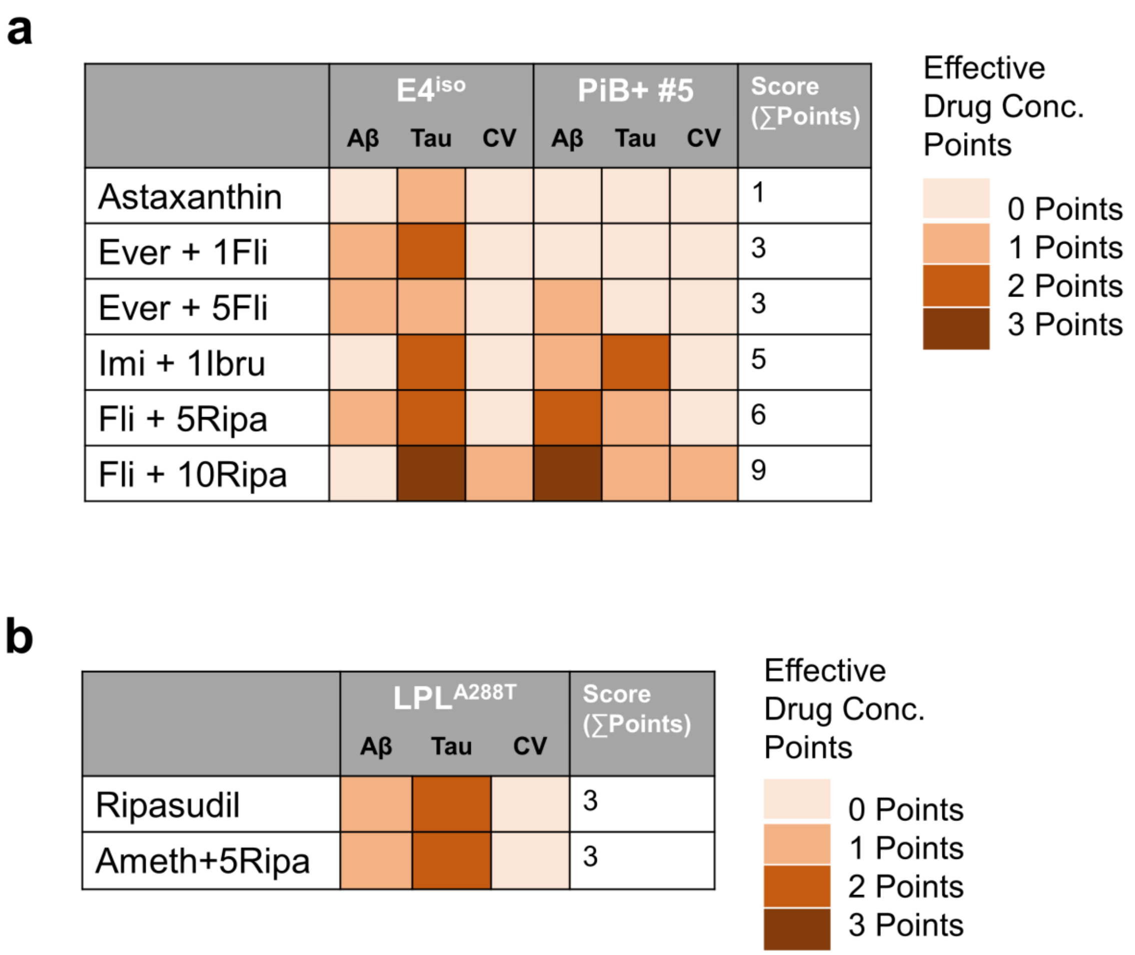

Supplementary Fig. 12. Scoring of the drug- effectiveness according to drug concentration points for the **(a)** E4 target drugs and **(b)** LPL target drugs. Ever, Everolimus; Fli, Flibanserin; Imi, Imipramine; Ibru, Ibrutinib; Ripa, Ripasudil; Ameth, Abemaciclib methanesulfonate; CV, cell viability; Conc., concentration.

Supplementary Table 1. Demographic data of iCOs

Line	Source	ApoE type	PiB positivity	age	sex
**PiB^- #1**	PBMC	E3/E3	Negative	58	M
**PiB^- #2**	PBMC	E3/E3	Negative	61	M
**PiB^- #3**	PBMC	E3/E3	Negative	74	M
**PiB^- #4**	PBMC	E3/E3	Negative	76	F
**PiB^- #5**	PBMC	E3/E3	Negative	76	F
**PiB^+ #1**	PBMC	E3/E3	Positive	75	F
**PiB^+ #2**	PBMC	E3/E3	Positive	76	F
**PiB^+ #3**	PBMC	E3/E3	Positive	76	F
**PiB^+ #4**	PBMC	E3/E4	Positive	73	F
**PiB^+ #5**	PBMC	E4/E4	Positive	70	F
**$E3^{par}/E4^{iso}$**	Fibroblast	E3/E3 to E4/E4	N/A	75	F

- PBMC, peripheral mononuclear cells

Supplementary Table 2. Predicted node activities of ApoE ε4 allele (APOE4) case and their experimental supporting evidences

SNP	Node name	Predicted activity	References
APO E4	SIRT1	Down	Theendakara V, Patent A, Peters Libeu CA, Philpot B, Flores S, Descamps O, Poksay KS, Zhang Q, Cailing G, Hart M, John V, Rao RV, Bredesen DE. Neuroprotective Sirtuin ratio reversed by ApoE4. Proc Natl Acad Sci U S A. 2013 Nov 5;110(45):18303-8. doi: 10.1073/pnas.1314145110. Epub 2013 Oct 21. PMID: 24145446
	a_secretase	Down	Huebbe P, Schaffer S, Jofre-Monseny L, Boesch-Saadatmandi C, Minihane AM, Müller WE, Eckert GP, Rimbach G. Apolipoprotein E genotype and alpha-tocopherol modulate amyloid precursor protein metabolism and cell cycle regulation. Mol Nutr Food Res. 2007 Dec;51(12):1510-7. doi: 10.1002/mnfr.200700194. PMID: 18030662
	ABCA7	Down	Tomioka M, Toda Y, Mañucat NB, Akatsu H, Fukumoto M, Kono N, Arai H, Kioka N, Ueda K. Lysophosphatidylcholine export by human ABCA7. Biochim Biophys Acta Mol Cell Biol Lipids. 2017 Jul;1862(7):658-665. doi: 10.1016/j.bbalip.2017.03.012. Epub 2017 Mar 31. PMID: 28373057
	AKT	Down	Zhao N, Liu CC, Van Ingelgom AJ, Martens YA, Linares C, Knight JA, Painter MM, Sullivan PM, Bu G. Apolipoprotein E4 Impairs Neuronal Insulin Signaling by Trapping Insulin Receptor in the Endosomes. Neuron. 2017 Sep 27;96(1):115-129.e5. doi: 10.1016/j.neuron.2017.09.003. PMID: 28957663
	Apoer2	Down	Xian X, Pohlkamp T, Durakoglugil MS, Wong CH, Beck JK, Lane-Donovan C, Plattner F, Herz J. Reversal of ApoE4-induced recycling block as a novel prevention approach for Alzheimer's disease. Elife. 2018 Oct 30;7:e40048. doi: 10.7554/eLife.40048. PMID: 30375977
	APP	Up	Huang YA, Zhou B, Wernig M, Südhof TC. ApoE2, ApoE3, and ApoE4 Differentially Stimulate APP Transcription and Aβ Secretion. Cell. 2017 Jan 26;168(3):427-441.e21. doi: 10.1016/j.cell.2016.12.044. Epub 2017 Jan 19. PMID: 28111074
	BACE1	Up	Hou X, Adeosun SO, Zhang Q, Barlow B, Brents M, Zheng B, Wang J. Differential contributions of ApoE4 and female sex to BACE1 activity and expression mediate Aβ deposition and learning and memory in mouse models of Alzheimer's disease. Front Aging Neurosci. 2015 Oct 31;7:207. doi: 10.3389/fnagi.2015.00207. PMID: 26582141
	BDNF	Down	Huang YA, Zhou B, Wernig M, Südhof TC. ApoE2, ApoE3, and ApoE4 Differentially Stimulate APP Transcription and Aβ Secretion. Cell. 2017 Jan 26;168(3):427-441.e21. doi: 10.1016/j.cell.2016.12.044. Epub 2017 Jan 19. PMID: 28111074
	DLK	Up	Huang YA, Zhou B, Wernig M, Südhof TC. ApoE2, ApoE3, and ApoE4 Differentially Stimulate APP Transcription and Aβ Secretion. Cell. 2017 Jan 26;168(3):427-441.e21. doi: 10.1016/j.cell.2016.12.044. Epub 2017 Jan 19. PMID: 28111074
	MKK7	Up	Huang YA, Zhou B, Wernig M, Südhof TC. ApoE2, ApoE3, and ApoE4 Differentially Stimulate APP Transcription and Aβ Secretion. Cell. 2017 Jan 26;168(3):427-441.e21. doi: 10.1016/j.cell.2016.12.044. Epub 2017 Jan 19. PMID: 28111074
	ERK	Up	Huang YA, Zhou B, Wernig M, Südhof TC. ApoE2, ApoE3, and ApoE4 Differentially Stimulate APP Transcription and Aβ Secretion. Cell. 2017 Jan 26;168(3):427-441.e21. doi: 10.1016/j.cell.2016.12.044. Epub 2017 Jan 19. PMID: 28111074
	GSK3b	Up	Zhao N, Liu CC, Van Ingelgom AJ, Martens YA, Linares C, Knight JA, Painter MM, Sullivan PM, Bu G. Apolipoprotein E4 Impairs Neuronal Insulin Signaling by Trapping Insulin Receptor in the Endosomes. Neuron. 2017 Sep 27;96(1):115-129.e5. doi: 10.1016/j.neuron.2017.09.003. PMID: 28957663
	CLU	Up	Jackson RJ, Rose J, Tulloch J, Henstridge C, Smith C, Spires-Jones TL. Clusterin accumulates in synapses in Alzheimer's disease and is increased in apolipoprotein E4 carriers. Brain Commun. 2019;1(1):fcz003. doi: 10.1093/braincomms/fcz003. Epub 2019 Jun 24. PMID: 31853523
	Reelin	Down	Lane-Donovan C, Herz J. The ApoE receptors Vldlr and Apoer2 in central nervous system function and disease. J Lipid Res. 2017 Jun;58(6):1036-1043. doi: 10.1194/jlr.R075507. Epub 2017 Mar 14. PMID: 28292942
	Dab1	Down	Lane-Donovan C, Herz J. The ApoE receptors Vldlr and Apoer2 in central nervous system function and disease. J Lipid Res. 2017 Jun;58(6):1036-1043. doi: 10.1194/jlr.R075507. Epub 2017 Mar 14. PMID: 28292942
	s_NMDAR	Down	Chen Y, Durakoglugil MS, Xian X, Herz J. ApoE4 reduces glutamate receptor function and synaptic plasticity by selectively impairing ApoE receptor recycling. Proc Natl Acad Sci U S A. 2010 Jun 29;107(26):12011-6. doi: 10.1073/pnas.0914984107. Epub 2010 Jun 14. PMID: 20547867
	TFEB	Down	Parcon PA, Balasubramaniam M, Ayyadevara S, Jones RA, Liu L, Shmookler Reis RJ, Barger SW, Mrak RE, Griffin WST. Apolipoprotein E4 inhibits autophagy gene products through direct, specific binding to CLEAR motifs. Alzheimers Dement. 2018 Feb;14(2):230-242. doi: 10.1016/j.jalz.2017.07.754. Epub 2017 Sep 22. PMID: 28945989
	LAMP2	Down	Parcon PA, Balasubramaniam M, Ayyadevara S, Jones RA, Liu L, Shmookler Reis RJ, Barger SW, Mrak RE, Griffin WST. Apolipoprotein E4 inhibits autophagy gene products through direct, specific binding to CLEAR motifs. Alzheimers Dement. 2018 Feb;14(2):230-242. doi: 10.1016/j.jalz.2017.07.754. Epub 2017 Sep 22. PMID: 28945989
	PIP2	Down	Zhu L, Zhong M, Elder GA, Sano M, Holtzman DM, Gandy S, Cardozo C, Haroutunian V, Robakis NK, Cai D. Phospholipid dysregulation contributes to ApoE4-associated cognitive deficits in Alzheimer's disease pathogenesis. Proc Natl Acad Sci U S A. 2015 Sep 22;112(38):11965-70. doi: 10.1073/pnas.1510011112. Epub 2015 Sep 8. PMID: 26372964
	synj1	Up	Zhu L, Zhong M, Elder GA, Sano M, Holtzman DM, Gandy S, Cardozo C, Haroutunian V, Robakis NK, Cai D. Phospholipid dysregulation contributes to ApoE4-associated cognitive deficits in Alzheimer's disease pathogenesis. Proc Natl Acad Sci U S A. 2015 Sep 22;112(38):11965-70. doi: 10.1073/pnas.1510011112. Epub 2015 Sep 8. PMID: 26372964
	PGC1a	Down	Yin J, Nielsen M, Carcione T, Li S, Shi J. Apolipoprotein E regulates mitochondrial function through the PGC-1α-sirtuin 3 pathway. Aging (Albany NY). 2019 Dec 6;11(23):11148-11156. doi: 10.18632/aging.102516. Epub 2019 Dec 6. PMID: 31808750
	PP2A	Down	Theendakara V, Bredesen DE, Rao RV. Downregulation of protein phosphatase 2A by apolipoprotein E: Implications for Alzheimer's disease. Mol Cell Neurosci. 2017 Sep;83:83-91. doi: 10.1016/j.mcn.2017.07.002. Epub 2017 Jul 15. PMID: 28720530
	PP2B	Up	Neustadtl AL, Winston CN, Parsadanian M, Main BS, Villapol S, Burns MP. Reduced cortical excitatory synapse number in APOE4 mice is associated with increased calcineurin activity. Neuroreport. 2017 Jul 5;28(10):618-624. doi: 10.1097/WNR.0000000000000811. PMID: 28542068

Supplementary Table 3. List of FDA-approved drugs and their corresponding molecular targets in the network model

Drug name	Original indication	Expected effects	References
**Everolimus**	Inhibitor of tumor cells proliferation, and activator of apoptosis and autophagy (Adult patients with progressive, well-differentiated non-functional, neuroendocrine tumors (NET) of gastrointestinal (GI) or lung origin)	mTOR inhibition	Du L, Li X, Zhen L, Chen W, Mu L, Zhang Y, Song A. Everolimus inhibits breast cancer cell growth through PI3K/AKT/mTOR signaling pathway. Mol Med Rep. 2018 May;17(5):7163-7169. doi: 10.3892/mmr.2018.8769. Epub 2018 Mar 16. PMID: 29568883
**Flibanserin**	Full agonist of the serotonin 5-HT1A receptor and 5-HT2A (Hypoactive sexual desire disorder in premenopausal women)	PTEN inhibition (PI3K-AKT signaling pathway)	Altieri SC, Garcia-Garcia AL, Leonardo ED, Andrews AM. Rethinking 5-HT1A receptors: emerging modes of inhibitory feedback of relevance to emotion-related behavior. ACS Chem Neurosci. 2013 Jan 16;4(1):72-83. doi: 10.1021/cn3002174. Epub 2012 Dec 20. PMID: 23336046
**Ripasudil**	Inhibitor of ROCK (Glaucoma and ocular hypertension)	Dkk1 inhibition (non-canonical WNT pathway)	Rao PV, Pattabiraman PP, Kopczynski C. Role of the Rho GTPase/Rho kinase signaling pathway in pathogenesis and treatment of glaucoma: Bench to bedside research. Exp Eye Res. 2017 May;158:23-32. doi: 10.1016/j.exer.2016.08.023. Epub 2016 Sep 1. PMID: 27593914
**A-meth.sulfonate**	Selective inhibitor of CDK4/6 (Breast cancer)	Cdk5 inhibition (Apoptosis- related pathway)	Yadav V, Burke TF, Huber L, Van Horn RD, Zhang Y, Buchanan SG, Chan EM, Starling JJ, Beckmann RP, Peng SB. The CDK4/6 inhibitor LY2835219 overcomes vemurafenib resistance resulting from MAPK reactivation and cyclin D1 upregulation. Mol Cancer Ther. 2014 Oct;13(10):2253-63. doi: 10.1158/1535-7163.MCT-14-0257. Epub 2014 Aug 13. PMID: 25122067
**Astaxanthin**	Potent antioxidant with antiproliferative neuroprotective and anti-inflammatory activity (Anti-aging ingredient)	TP53 inhibition (Apoptosis- related pathway)	Wang XJ, Chen W, Fu XT, Ma JK, Wang MH, Hou YJ, Tian DC, Fu XY, Fan CD. Reversal of homocysteine-induced neurotoxicity in rat hippocampal neurons by astaxanthin: evidences for mitochondrial dysfunction and signaling crosstalk. Cell Death Discov. 2018 Oct 22;4:50. doi: 10.1038/s41420-018-0114-x. Erratum in: Cell Death Discov. 2019 Mar 1;5:70. Erratum in: Cell Death Discov. 2019 Jul 10;5:116. PMID: 30374413
**Ibrutinib**	Selective irreversible inhibitor of BTK (Adult patients with chronic graft-versus-host disease (cGVHD))	MKK7 inhibition (APOE4-MAPK pathway)	Shraga A, Olshvang E, Davidzohn N, Khoshkenar P, Germain N, Shurrush K, Carvalho S, Avram L, Albeck S, Unger T, Lefker B, Subramanyam C, Hudkins RL, Mitchell A, Shulman Z, Kinoshita T, London N. Covalent Docking Identifies a Potent and Selective MKK7 Inhibitor. Cell Chem Biol. 2019 Jan 17;26(1):98-108.e5. doi: 10.1016/j.chembiol.2018.10.011. Epub 2018 Nov 15. PMID: 30449673
**Imipramine**	Inhibitor of serotonin transporter (Major depressive disorder)	synj1 inhibition (Synapse loss related pathway)	Wong ML, O'Kirwan F, Hannestad JP, Irizarry KJ, Elashoff D, Licinio J. St John's wort and imipramine-induced gene expression profiles identify cellular functions relevant to antidepressant action and novel pharmacogenetic candidates for the phenotype of antidepressant treatment response. Mol Psychiatry. 2004 Mar;9(3):237-51. doi: 10.1038/sj.mp.4001470. PMID: 14743185

Supplementary Table 4. List of primers used in the study

Gene	Primer sequence
hSOX2	5’-GGG AAA TGG GAG GGG TGC AAA AGA GG-3’ (forward)<br>5’-TTG CGT GAG TGT GGA TGG GAT TGG TG-3’ (reverse)
hOct4	5’-GGA GGA AGC TGA CAA CAA TGA AA C-3’ (forward)<br>5’-GGC CTG CAC GAG GGT TT-3’ (reverse)
hGAPDH	5’-GTG GAC CTG ACC TGC CGT CT-3’ (forward)<br>5’-GGA GGA GTG GGT GTC GCT GT-3’ (reverse)

Supplementary Methods

Generation, maintenance, and characterization of human iPSCs

To generate human iPSCs from patient-derived peripheral blood mononuclear cells (PBMCs), we adopted a previously published protocol[1]. Whole-blood samples were obtained via venipuncture in K2 ethylenediaminetetraacetic acid (EDTA) tubes (367525, BD Biosciences). PBMCs were separated by density gradient centrifugation with Ficoll Paque Plus (17-1440-03, GE Healthcare), washed with PBS, centrifuged at 300 g for 15 minutes, and resuspended in KBM502 medium (16025020, Kohjin Bio) at 1 x 10^6 cells per mL. Cells were plated at a density of 5 x 10^5 cells per well of a 24-well plate pre-coated with human CD3-specific antibody (10 µg/ml; 555336, BD Biosciences) and incubated at 37°C in 5% CO2 for 5 days, with addition of fresh KBM502 medium on the second day. On the fifth day, activated T cells were collected, resuspended in fresh KBM502 medium, and infected with Sendai virus using a CytoTune-iPS 2.0 Sendai Reprogramming Kit (A16517, Invitrogen) at 8 MOI per 5 x 10^5 cells. The cells were then re-plated on a new CD3 antibody-coated plate. On the sixth day, infected T cells were collected, resuspended in mTeSR1 medium (ST85850, Stemcell Technologies) and plated on a 100-pi dish coated with Matrigel hESC-qualified Matrix (354277, Corning). Two days after the cells were plated, the medium was changed with fresh mTeSR1 medium. Thereafter, the medium was changed daily. iPSC colonies began to appear on days 15-20 and were manually picked. The cells were passaged every 5 days on average, using ReLeSR (ST05872, Stemcell Technologies) as a detachment reagent. For the characterization of iPSCs, karyotype analysis was performed at GenDix, Inc. (Seoul, South Korea), and alkaline phosphatase (ALP) staining was conducted by using an ALP detection kit (SCR004, Millipore) according to the manufacturer's instructions. ApoE ε3 parental and ApoE ε4 isogenic iPSC lines had been used and proven in previous papers[2, 3].

Generation of iCOs from iPSCs

To form embryoid bodies (EBs), hiPSCs maintained on Matrigel-coated plates were detached with ReLeSR and intact colonies were dissociated to single cells in AggreWell EB Formation Medium (ST05893, Stemcell Technologies) supplemented with the ROCK inhibitor, Y-27632 (ST72304, Stemcell Technologies). On Day 0, 2 x 10^6 cells per well were loaded to an AggreWell800 24-well plate (34811, Stemcell Technologies); on the following day, the medium was replaced with EB Formation Medium lacking Y27632. Thereafter, we referred to the previously published protocol for generation of iCOs[2, 4, 5] and optimized for culture on a 96-well plate for drug screening. In detail, from Day 2 to Day 5, the medium was replaced daily with DMEM/F-12 containing GlutaMAX (10565-018, Gibco), 20% KnockOut Serum Replacement (A3181501, Gibco), 1% MEM Non-Essential Amino Acids Solution (11140050, Gibco), 0.1 mM 2-mercaptoethanol (21985023, Gibco), and 100 U/ml penicillin and 100 µg/ml streptomycin (P4333, Merck), and further supplemented with the SMAD inhibitors, dorsomorphin (10 µM; P5499, Merck) and SB-431542 (10 µM; 1614, TOCRIS). On Day 6,

EBs were collected; on the following day, individual EBs were seeded to individual wells of a 96-well ultra-low-attachment microplate (Corning, 7007). Organoids were cultured in neural medium composed of Neurobasal-A Medium (10888-022, Gibco), B-27 Supplement minus vitamin A (12587010, Gibco), 100 U/ml penicillin and 100 μg/ml streptomycin, GlutaMAX (35050-061, Gibco), and 0.5%(v/v) Matrigel Basement Membrane Matrix (354234, Corning). From Day 6 to Day 15, daily medium changes were performed with neural medium supplemented with 20 ng/ml Epidermal Growth Factor (EGF; 01-107, Merck) and 20 ng/ml Fibroblast Growth Factor basic (bFGF; 233-FB, R&D Systems). From Day 16 to Day 24, the medium was changed every other day using the same medium. From Day 25 to 42, the medium was changed every other day using the neural medium in which the EGF and bFGF were replaced with 20 ng/ml Brain Derived Neurotrophic Factor (BDNF; 450-02, Peprotech) and 20 ng/ml Neurotrophin-3 (NT-3; 450-03, Peprotech). From Day 43 onwards, the medium was changed every 4 days using neural medium without any growth factor.

Immunocytochemistry

iPSCs were cultured on slide glasses coated with Matrigel hESC-qualified Matrix. For experiments, the medium was aspirated and the cells were washed with PBS, fixed with 4% paraformaldehyde (PFA) in PBS for 20 minutes at RT, washed with PBS, and blocked with 0.3% Triton X-100 (X100, Merck) in PBS (PBST) containing 5% bovine serum albumin (BSA; 10857, Affymetrix) for 2 hours at RT. Primary antibodies were diluted in 0.3% PBST containing 1% BSA and applied to the fixed cells at 4°C overnight. The antibodies detected Oct4 (1:1000) and Tra1-60 (1:500), and came from the human Embryonic Stem Cell Marker Panel (ab109884, Abcam). The slides were washed with PBS and incubated for 1 hour at RT in a secondary antibody solution consisting of 0.3% PBST, 1% BSA, and Alexa Fluor-conjugated secondary antibodies (1:500). The slides were washed with 0.3% PBST, nuclei were stained with DAPI for 10 minutes at RT, and the slides were washed with PBS, dried, and mounted for confocal imaging.

Immunohistochemistry

Organoids were washed with PBS, immersed in 4% PFA at 4°C overnight, washed to remove the remaining PFA, immersed in 30% sucrose at 4°C for 72 hours, and then moved to a cryomold and frozen in FSC 22 Compound (3801480, Leica). Organoids were cryosectioned and the slices were washed with PBS and permeabilized using 0.3% Triton X-100 (X100, Merck) in PBS for 30 minutes at room temperature (RT). Slices were blocked using 5% normal horse serum (S-2000, Vector Laboratories) in PBS for 1 hour at RT. Primary antibodies were diluted in blocking solution and applied at 4°C overnight. Fluorophore-conjugated secondary antibodies were diluted at ratio of 1:1000 in 3% BSA in PBS and treated for 1 hour at RT. Slices were washed, stained with DAPI, and mounted on slides. Confocal images were acquired using LSM700 Laser Confocal Microscope (Zeiss). The following antibodies were used: NeuN (1:500; 24307, Cell Signaling Technology), beta-III tubulin (1:500; G7121, Promega), SOX2 (1:500; GT15098, Neuromics), MAP2 (1:500; ab5392, Abcam).

Drug treatment

Organoids cultured on a 96-well plate were washed with Opti-MEM Reduced Serum Medium (31985-070, Gibco) containing 100 U/ml penicillin and 100 µg/ml streptomycin (P4333, Merck). For treatment, the drug concentrate was mixed in the medium and applied to the organoids at 37°C for 24 hours. The following drugs were used with concentration ranges indicated in parentheses: Astaxanthin (0.125–5 µM; SML0982, Merck), Everolimus (0.25–5 µM; SML2282, Merck), Flibanserin (1–20 µM; HY-A0095, MedChemExpress), Imipramine hydrochloride (0.25–20 µM; I7379, Merck), Ibrutinib (1 µM; HY-10997, MedChemExpress), Ripasudil (0.25–10 µM; HY-15685, MedChemExpress), and Abemaciclib methanesulfonate (0.25–2 µM; HY-16297, MedChemExpress). In comparative experiments, no drug treatment groups (called as vehicle group; Veh) were used as control groups. In other words, since our drug powders were initially diluted in DMSO or distilled water (DW) to make them dissolve, iCOs of Veh groups received a treatment with appropriate amount of DMSO or distilled water (DW) (the same volume of solution used to make drug-powders dissolve) that do not contain drugs meant to affect iCOs. Also, six replicates (individual iCOs) per each drug-dose were used for the experiments. Detailed information on concentrations is shown in Fig. 6.

Reverse transcriptase quantitative PCR (RT-qPCR)

All steps were performed as described by the manufacturers' protocols. Briefly, total RNA was isolated with a RNeasy Plus Mini Kit (74136, QIAGEN), cDNA was synthesized with a Maxime RT PreMix Kit (25081, iNtRON Biotechnology), and real-time quantitative PCR (qPCR) was performed using the SYBR Green Fast mix (KR0389, KABA biosystems). Target sequences were amplified using primers listed on Supplementary Table 4. Expression levels of mRNAs were calculated using the ΔΔCt of each target mRNA. The expression level of human GAPDH, a housekeeping gene, was used as an endogenous control for each sample.

RNA sequencing, differentially expressed genes (DEGs), and Gene Ontology (GO) analysis

Total RNA was extracted from organoids with or without aging effect (MST-312, telomerase inhibitor, M3949, Sigma) using Ribospin 2 (314-150, GeneAll Biotechnology), and cDNA was synthesized with a TruSeq Stranded Total RNA Sample Prep Kit (Illumina). In brief, ribosomal RNA was depleted and the remaining RNA was fragmented using divalent cations. First-strand cDNA was made from RNA fragments using reverse transcriptase and random primers, and second-strand cDNA synthesis was performed using DNA Polymerase I and RNase H. The products were further purified, PCR amplified, and sequenced on a NovaSeq6000 (Illumina) platform. For analysis, genome indexes were generated using GRCh37.p13 GENCODE v19 annotation, and FASTQ files were transformed to BAM files using the STAR-2.7.1a software. RNA sequencing data is available at NCBI under SRA accession number PRJNA678865 (https://www.ncbi.nlm.nih.gov/bioproject/678865).

To demonstrate that gene expression profiles differ between PiB^- (or $E3^{par}$) iCOs and PiB^+ (or

E4^{iso}) iCOs (Fig 2i and Supplementary Fig 2a, c, f, 3), DEGs were defined by the following steps. First, count matrixes were loaded, and sample information and gene annotation were organized using the readDGE function offered by edgeR. Second, data were pre-processed by filtering out genes with low-level expression (filterByExpr function with default option) and performing TMM-based normalization (calcNormFactors function with method TMM), using edgeR in both cases. Finally, differential expression analysis was performed. The 'design' matrix and 'contrast' were constructed using the model.matrix and makeContrasts functions, respectively. The voom, limFit, contrasts.fit, and eBayes functions were run sequentially to remove heteroscedasticity from count data. The topTable function was used to list DEGs (cutoff, $\log_2$(fold change) <-1 or >1 and $-\log_{10}$(p-value) >1). All functions were provided by limma. The DEGs from the RNA sequencing data were re-analyzed using the Toppgene database (https://toppgene.cchmc.org/). Transcriptomic GO analyses between the PiB$^-$ iCOs and PiB$^+$ iCOs or E4^{iso} iCOs and E3^{par} iCOs were performed with the FDR-corrected p-value < 0.05. The relevant sub-ontologies were molecular function (MF), biological process (BP), cellular components (CC), and the disease. In addition, GO similarity analysis was also performed to identify how many GO terms overlapped with public GO data from the Gene Expression Omnibus (GEO) database (Accession number: GSE143951, GSE109887; Platform number: GPL16043, GPL10904)[6, 7]. To analyze public transcriptome data, GEO2R analyzer (https://www.ncbi.nlm.nih.gov/geo/geo2r) was used.

Independently, the DEGs for the validation of mathematical model and identification of individual drug target genes (Fig. 5b and Supplementary Fig. 8, 9) were obtained with FDR-corrected p-value < 0.05 and $\log_2$(fold-change) > 1 or < -1, using DEGseq package in R. Furthermore, pathway enrichment analysis was carried out following the DEG analysis.

Ethyl-cinnamate (ECi) 3D tissue clearing

We followed a previously published protocol[8] with minor adjustments for use with a 96-well plate. Organoids were washed with PBS, fixed in 4% PFA at 4°C overnight, and washed in PBS three times to remove residual formaldehyde. For blocking and permeabilization, organoids were shaken at RT for 2 days in PBS-TxDBN solution [2% Triton-X-100 (X-100, Sigma), 20% Dimethyl Sulfoxide (DMSO; D4540, Sigma), 5% BSA, 0.05% NaN_3 in PBS]. Organoids were stained with primary antibodies in 96-well low-attachment plates (7007, Corning) in 200 µl PBS-TxDBN/well at RT for 5 days on an orbital shaker spinning at 280 rpm. Organoids were subsequently washed with PBS-TxDBN for 2 days. Secondary antibody staining was performed as described for the primary antibody. Stained organoids were washed and fixed with 4% paraformaldehyde in PBS at 4°C overnight. They were dehydrated sequentially into a series of 30%, 50%, 70%, and 2 x 99.7% 1-propanol (anhydrous; 279544, Sigma) in PBS, with pH adjusted to 9.0-9.5 using trimethylamine. Each dehydration step was performed for at least 4 hours at 4°C. After dehydration, organoids were immersed in ethyl cinnamate (112372, Sigma) for index matching and incubated at room temperature for 1 hour before imaging. The following antibodies were used: β-amyloid (D54D2) (1:50; 8243, Cell Signaling Technology), phospho-Tau (Thr181) (1:100; MN1050, Thermo Fisher), and Alexa

Fluor-conjugated secondary antibodies (1:150).

Calcium oscillation analysis with the FLIPR Calcium 6 assay

To evaluate the calcium flux in organoids, we used a FLIPR Calcium 6 Assay Kit (R8290, Molecular Devices) according to the manufacturer's protocol. Organoids were plated to microplates in 100 µl culture medium per well and incubated overnight at 37°C in 5% CO_2. A vial of Calcium 6 Assay Reagent (Component A) was equilibrated to room temperature and thoroughly mixed with 10 mL Component B (1X Hank's Balanced Salt solution plus 20 mM HEPES buffer, pH 7.4). An equal volume of loading buffer (100 µl) was added to each well of the 96-well microplate, which was incubated in 37°C in 5% CO_2 incubator for 2 hours. The fluorescent signal was detected using an ImageXpress Micro Confocal High-Content Imaging System (Molecular Devices).

Input-output relationships of the network model

We validated the dynamics of our network model by analyzing its input-output relationships[9]. To analyze the response characteristics of output nodes (Aβ, p-tau, autophagy, apoptosis and synapse loss) to the input node stimulation representing aging effects (such as oxidative stresses) for each condition (Normal, ApoE ε4 allele, and LPL SNP cases), we measured the activity change of output nodes along with the increase of the input node activity. Here, we defined the activity of a node as the average of 'ON' state ratio over the last 300 time-steps of simulation out of 1,000 time-steps as these were sufficient enough to converge to steady-states. For instance, if the state of a node was observed as '0101010101', the steady-state activity of the node is determined to be 50% ON. Similarly, the input intensity was defined by the average ON state during the simulation. We increased the input intensity from 0 to 100% ON with 1% interval, and measured the steady-state activity of the output nodes. We have further performed initial condition sensitivity analysis of the input-output analyses. From the total of 77 nodes, a certain number of nodes except 'ROS', 'APOE4' and 'LPL' have been randomly selected and given an initial state value of '1' and the others have been set to '0'. The number of selected nodes has been set from 10 to 70 with 10 nodes interval, and the input-output analysis has been performed for these randomly generated initial state values. The average node activities obtained from this analysis have been compared with the previous results using the 'pcc' function of the 'sensitivity' R package. We have set the value of the 'nboot' parameter of the function to 100. The results of this initial condition sensitivity analysis of our Boolean model have all showed Pearson correlation coefficients of 1. This is because, when we fixed the value of 'APOE4' or 'LPL' node for given genetic conditions, all initial states of each simulation trial have converged to only one attractor. Consequently, we have confirmed that the results of input-output analyses are not affected by initial condition.

Supplementary References

1. Seki T, Yuasa S, Fukuda K. Generation of induced pluripotent stem cells from a small amount of human peripheral blood using a combination of activated T cells and Sendai virus. *Nat Protoc* **7**, 718-728 (2012).

2. Lin YT*, et al.* APOE4 Causes Widespread Molecular and Cellular Alterations Associated with Alzheimer's Disease Phenotypes in Human iPSC-Derived Brain Cell Types. *Neuron* **98**, 1141-1154 e1147 (2018).

3. Meyer K*, et al.* REST and Neural Gene Network Dysregulation in iPSC Models of Alzheimer's Disease. *Cell Rep* **26**, 1112-1127 e1119 (2019).

4. Pasca AM*, et al.* Functional cortical neurons and astrocytes from human pluripotent stem cells in 3D culture. *Nat Methods* **12**, 671-678 (2015).

5. Choi H*, et al.* Acetylation changes tau interactome to degrade tau in Alzheimer's disease animal and organoid models. *Aging Cell* **19**, e13081 (2020).

6. Martins S*, et al.* IPSC-Derived Neuronal Cultures Carrying the Alzheimer's Disease Associated TREM2 R47H Variant Enables the Construction of an Abeta-Induced Gene Regulatory Network. *Int J Mol Sci* **21**, (2020).

7. Lardenoije R*, et al.* Alzheimer's disease-associated (hydroxy)methylomic changes in the brain and blood. *Clin Epigenetics* **11**, 164 (2019).

8. Masselink W*, et al.* Broad applicability of a streamlined ethyl cinnamate-based clearing procedure. *Development* **146**, (2019).

9. Helikar T, Konvalina J, Heidel J, Rogers JA. Emergent decision-making in biological signal transduction networks. *Proc Natl Acad Sci U S A* **105**, 1913-1918 (2008).
