# Supplementary material for: A logical network-based drug-screening platform for Alzheimer’s disease representing pathological features of human brain organoids
Source: Nat Commun. 2021 Jan 12;12:280. doi: 10.1038/s41467-020-20440-5 (PMC7804132; doi:10.1038/s41467-020-20440-5)
Supplement: Supplementary file 5 — Description of Additional Supplementary Files [file 41467_2020_20440_MOESM5_ESM.pdf]

Title: Supplementary Data 1.
Description: Network model logic table (Annotation: | OR, & AND, ! NOT)
